# Maternal Gestational Diabetes Impairs Fetoplacental Insulin-Induced Vasodilation via AKT/eNOS Pathway and Reduces Placental Efficiency

**DOI:** 10.3390/ijms262311507

**Published:** 2025-11-27

**Authors:** Clara M. Hengst, Maria de Leyre Villar-Ballesteros, Heike Brendel, Sindy Giebe, Coy Brunssen, Alexander Frühauf, Cahit Birdir, Paul D. Taylor, Lucilla Poston, Henning Morawietz

**Affiliations:** 1 Division of Vascular Endothelium and Microcirculation, Department of Medicine III, Faculty of Medicine and University Hospital Carl Gustav Carus, TUD Dresden University of Technology, 01307 Dresden, Germany; clara.hengst@mailbox.tu-dresden.de (C.M.H.); leyre.villar@ukdd.de (M.d.L.V.-B.); coy.brunssen@ukdd.de (C.B.); 2 Department of Women & Children’s Health, School of Life Course Sciences, King’s College London, London SE1 7EH, UK; paul.taylor@kcl.ac.uk (P.D.T.);; 3 Department of Obstetrics and Gynecology, Center for Feto/neonatal Health, Faculty of Medicine and University Hospital Carl Gustav Carus, TUD Dresden University of Technology, 01307 Dresden, Germany

**Keywords:** cardiovascular diseases, endothelial dysfunction, gestational diabetes mellitus, insulin resistance, offspring, placenta

## Abstract

Gestational Diabetes Mellitus (GDM) increases the long-term risk for metabolic and cardiovascular diseases in the offspring. However, the underlying mechanisms are not well understood. This study investigates the impact of GDM on fetoplacental vascular function and molecular mechanisms underlying endothelial dysfunction. Clinical data and tissue samples were collected from normoglycemic (NG, n = 33) and GDM (n = 19) pregnancies. Offspring in the GDM group were delivered earlier, had a larger placental size, and had a reduced placental efficiency. Functional analysis using a Mulvany myograph demonstrated a significant impairment of insulin-mediated vasodilation in fetoplacental vessels of GDM patients compared to NG controls. This vascular dysfunction was associated with a reduction in total insulin receptor protein expression. Further investigation revealed an impaired PI3K/AKT/eNOS signaling pathway, as endothelial cells from GDM pregnancies showed a deficient insulin-induced phosphorylation of AKT. These results indicate that maternal GDM induces insulin resistance and endothelial dysfunction in the fetoplacental vasculature through impairment of the AKT/eNOS pathway, providing a key mechanism for its adverse neonatal outcomes and the increased lifelong cardiovascular risk in the offspring.

## 1. Introduction

Gestational Diabetes Mellitus (GDM) is defined as hyperglycemia first recognized during pregnancy and typically detected in the second or third trimester of pregnancy [1]. The prevalence of GDM has been constantly increasing in recent years [2,3]. Given the significant health risks it poses to mother and offspring, its global importance becomes evident [4]. GDM increases the risk of adverse pregnancy outcomes, such as preeclampsia or acute respiratory distress syndrome (ARDS), and contributes to the development of long-term complications, including obesity, cardiovascular disease (CVD), and insulin resistance, in both mother and child [5]. The association between GDM and the increased cardiovascular risk in the offspring has been well supported by large population-based studies. Pathirana et al. demonstrate that children exposed to GDM exhibit higher systolic blood pressure compared to controls [6], which can contribute to a 2-fold increased risk of developing CVD in GDM offspring [7]. Moreover, the offspring of mothers with gestational and pregestational diabetes show a higher risk of developing cardiovascular complications such as heart failure and pulmonary embolism at early adulthood [8].

There is growing evidence that placental dysfunction plays a crucial role in the fetal programming of CVD in the offspring [9,10,11]. The placenta has a dense network of fetal blood vessels enabling the exchange between fetal and maternal circulation and mediating metabolic, excretory, endocrine and immunologic functions [12]. Its vasculature is essential for an adequate oxygen and nutrient delivery, which is crucial for healthy fetal growth and development. In contrast, GDM can induce endothelial dysfunction not only in women with a history of GDM and their offspring but also in the umbilical–placental circulation [13]. Previous studies have demonstrated impaired endothelium-dependent relaxation in the mesenteric arteries of rat offspring [14,15], as well as impaired endothelium-dependent vasorelaxation in GDM umbilical veins in response to calcitonin [16]. One underlying mechanism might be the development of vascular insulin resistance as a key feature of diabetes, characterized by impaired insulin-induced vasodilation leading to endothelial dysfunction. Under healthy conditions, insulin promotes vasodilation by activating the PI3K/AKT pathway. This results in phosphorylation of the endothelial nitric oxide synthase (eNOS) at Ser1177, leading to increased nitric oxide (NO) production [17] as a critical mediator of vascular relaxation. In contrast, hyperinsulinemia [18] leads to insulin resistance, impairs the pathway, and reduces eNOS activation [19]. This results in diminished NO production and impaired vasodilation, promoting the development of CVD.

Understanding the mechanisms underlying the impaired insulin-induced vasodilation is critical to elucidate its role in CVD development [20]. In GDM, disrupted insulin signaling in the placenta may impair placental vascular adaptation, exacerbate adverse effects on the fetus and increase the long-term cardiovascular risk in the offspring. This study aims to investigate the impact of GDM on the fetoplacental vascular function and to identify key molecular pathways involved in placental vascular insulin resistance. This might provide potential therapeutic targets in the prevention of cardiovascular complications of GDM.

## 2. Results

### 2.1. Maternal, Neonatal, and Placental Outcomes Associated with GDM

The analysis of clinical data collected from mothers and their offspring is summarized in Table 1. Maternal data confirm the correct classification of the two study groups based on the Oral Glucose Tolerance Test (OGTT) results, with the GDM group exhibiting significantly elevated glycemia at both 1 h and 2 h post-glucose ingestion. Additionally, patients in the GDM group had a higher body mass index compared to the normoglycemic (NG) group. In addition, we performed an exploratory sex-stratified analysis (Supplementary Table S1) and observed that GDM mothers carrying female fetuses had a significantly higher BMI compared with normoglycemic mothers. Moreover, all women in the GDM group delivered by cesarean section, a rate that was significantly higher compared to the NG group, highlighting the increased risk of surgical delivery associated with GDM.

**Figure 1:**
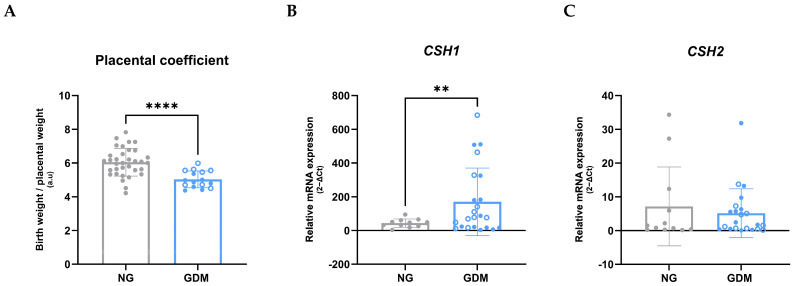
**Offspring from GDM mothers shows early signs of placental dysfunction.** (**A**) Placental coefficient is significantly decreased in pregnancies with GDM compared to NG controls (NG = 6.05 ± 0.81; GDM = 5.03 ± 0.48; *p* < 0.0001. (**B**) Relative mRNA expression of *CSH-1* in fetoplacental vessels is significantly upregulated in GDM pregnancies (NG = 43.48 ± 24.05; GDM = 169.80 ± 195.48; *p* = 0.0066). (**C**) Relative mRNA expression of *CSH-2* in fetoplacental vessels is unchanged between NG and GDM pregnancies (NG = 7.16 ± 11.19; GDM = 5.14 ± 7.10; *p* = 0.5928). Data are presented as mean ± standard deviation. Statistical significance was assessed using Welch’s *t*-test, with *p* < 0.05 considered statistically significant. Asterisks indicate significance levels: ** *p* < 0.01, **** *p* < 0.0001. Samples sizes were n_NG_ = 34 and n_GDM_ = 16 (**A**), n_NG_ = 11 and n_GDM_ = 23 (**B**), n_NG_ = 12 and n_GDM_ = 22 (**C**) (with dGDM samples shown as empty dots and iGDM as filled dots). *CSH-1* and *CSH-2*, chorionic somatomammotropin hormone 1 and 2, GDM, gestational diabetes mellitus; NG, normoglycemic group.

In neonates, a significant reduction in the Apgar score (activity, pulse, grimace, appearance, respiration) at 5 min after birth was observed in the GDM group. Although birth weights did not differ significantly between the NG and GDM groups, neonates in the GDM group were delivered significantly earlier, as indicated by gestational age in weeks. Moreover, the placental size was increased in the GDM group, evidenced by greater placental weight, width, length, and surface area, while umbilical cord size remained unchanged. Analysis of the placental coefficient, defined as the ratio of neonatal birth weight to placental weight and used as an indicator of placental efficiency, revealed a significant decrease in the GDM group (Figure 1A). Furthermore, mRNA expression analysis of chorionic somatomammotropin hormone 1 (*CSH-1*) and 2 (*CSH-2*) in fetoplacental vessels, markers of placental hormone production and function, demonstrated a significant upregulation of *CSH-1* (Figure 1B) in GDM patients, whereas *CSH-2* expression showed no significant differences (Figure 1C).

### 2.2. GDM Impairs Insulin-Mediated Vasodilation in Fetoplacental Vessels

**Figure 2:**
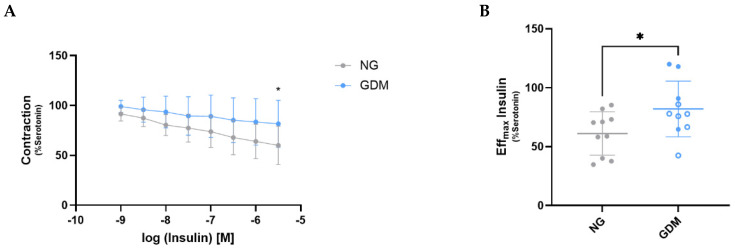
**Maternal GDM reduces insulin-induced vasorelaxation in fetoplacental vessels.** (**A**) Dose–response curve showing insulin-induced vasorelaxation in vessels from NG and GDM pregnancies. GDM vessels exhibited significantly reduced relaxation at the highest insulin concentration tested. (**B**) Quantification of vessel relaxation at the maximal insulin concentration revealed significantly increased relaxation in NG vessels compared to GDM (NG = 61.15 ± 17.48%; GDM = 82.01 ± 22.37%; *p* = 0.0415). Data are shown as mean ± standard deviation. Statistical significance was assessed using two-way repeated measures ANOVA (**A**) and Welch’s *t*-test (**B**), with *p* < 0.05 considered statistically significant, indicated by an asterisk (*). Samples sizes were n_NG_ = 10 and n_GDM_ = 10 (with n_dGDM_ = 6 shown as empty dots and n_iGDM_ = 4 as filled dots). GDM, gestational diabetes mellitus; NG, normoglycemic group.

**Figure 3:**
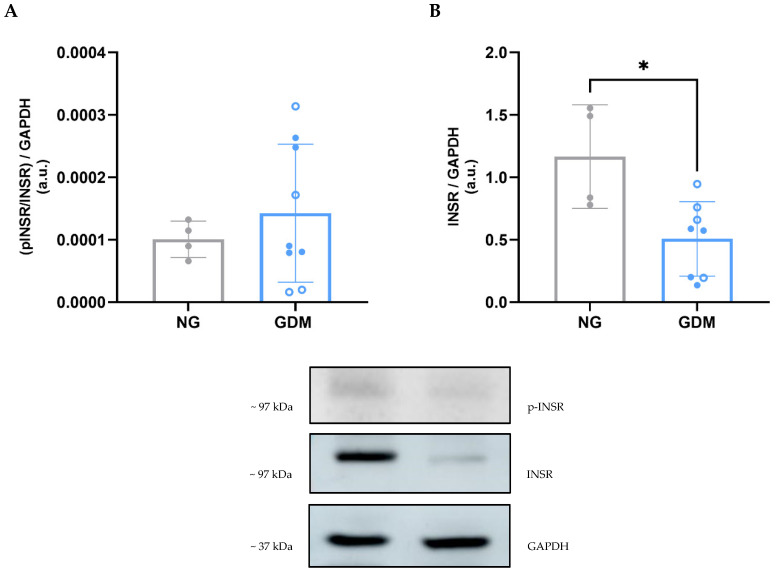
**Effect of maternal GDM on INSR expression and activation in fetoplacental vessels.** (**A**) INSR activation, measured as the ratio of p-INSR/INSR, was not different between the two groups (NG = 1.01 × 10^−4^ ± 1.59 × 10^−5^; GDM = 1.26 × 10^−4^ ± 9.52 × 10^−5^; *p* = 0.5649). (**B**) INSR expression was significantly decreased in the GDM group compared to NG (NG = 1.17 ± 0.31; GDM = 0.51 ± 0.28; *p* = 0.0398). Data are presented as mean ± standard deviation. Statistical significance was determined using Welch’s *t*-test, with *p* < 0.05 considered statistically significant, indicated by an asterisk (*). Samples sizes were n_NG_ = 4 and n_GDM_ = 8 (with n_dGDM_ = 4 shown as empty dots and n_iGDM_ = 4 as filled dots). GAPDH, glyceraldehyde-3-phosphate dehydrogenase, GDM, gestational diabetes mellitus; INSR, insulin receptor; NG, normoglycemic group; p-INSR, phosphorylated insulin receptor.

Both NG and GDM fetoplacental vessels were subjected to functional analysis using a Mulvany myograph to evaluate their vasodilatory response to insulin. Serotonin-precontracted fetal vessels exhibited insulin-induced vasorelaxation (Figure 2A), with NG vessels showing a significantly greater vasodilatory response at the highest insulin concentration tested (Figure 2B). These findings suggest an impairment in the insulin signaling pathway in GDM vessels. Consequently, we investigated potential alterations in insulin receptor (INSR) expression and activation in these fetoplacental vessels. Western blot analysis revealed no significant differences in INSR phosphorylation at Tyr1150/1151 between the two groups (Figure 3A), but total INSR expression was reduced in GDM vessels compared to controls (Figure 3B).

**Figure 4:**
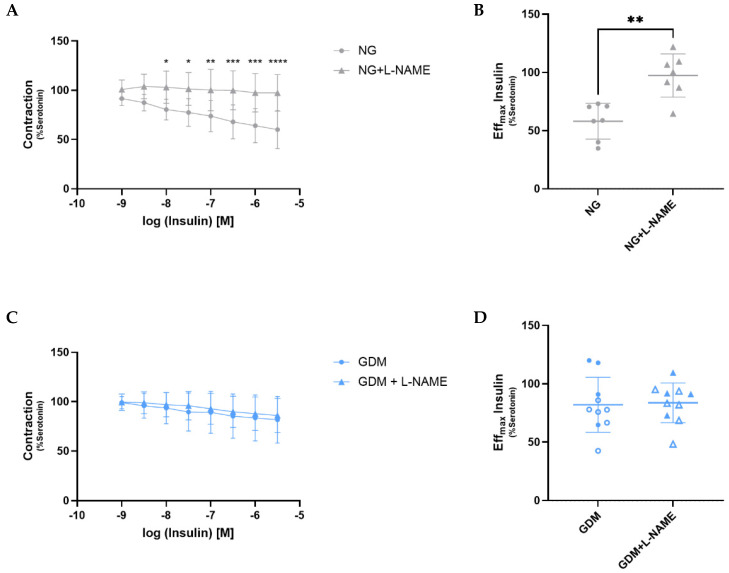
**Insulin-dependent vasorelaxation is NO-independent in GDM fetoplacental vessels.** (**A**) L-NAME significantly reduced insulin-mediated relaxation in NG vessels at different insulin concentrations. (**B**) At maximum insulin concentration, NG + L-NAME vessels showed decreased relaxation (NG = 58.07 ± 14.20%; NG + L-NAME = 97.37 ± 17.14%; *p* = 0.0062). (**C**) L-NAME did not affect insulin-mediated relaxation in GDM vessels. (**D**) At maximum insulin concentration, no differences in relaxation between GDM and GDM + L-NAME vessels were observed (GDM = 82.01 ± 22.37%; GDM + L-NAME = 83.64 ± 16.18%; *p* = 0.7209). Data are presented as mean ± standard deviation. Statistical significance was assessed using two-way repeated measures ANOVA (**A**,**C**) and paired *t*-test (**B**,**D**), with *p* < 0.05 considered statistically significant. Asterisks indicate significance levels: * *p* < 0.05, ** *p* < 0.01, *** *p* < 0.001, **** *p* < 0.0001. Samples sizes were n_NG_ = 7 and n_GDM_ = 10 (with n_dGDM_ = 6 shown as empty symbols and n_iGDM_ = 4 as filled symbols). GDM, gestational diabetes mellitus; L-NAME, L-N^G^-Nitro arginine methyl ester; NG, normoglycemic group; NO, nitric oxide.

To further explore the insulin-induced, NO-dependent vasorelaxation capacity of the fetoplacental vessels, we performed Mulvany myograph experiments in the presence of the nitric oxide synthase (NOS) inhibitor L-NAME. This allowed us to assess vasodilation independent of NO signaling. NG vessels exhibited a significant reduction in insulin-induced vasodilation after L-NAME treatment (Figure 4A,B), indicating that their vasodilatory response is largely NO-dependent. In contrast, GDM vessels showed no significant difference in vasodilation with or without L-NAME (Figure 4C,D), suggesting that insulin-induced vasodilation in GDM vessels is largely independent of endothelial nitric oxide synthase (eNOS) activity.

### 2.3. Disruption of the AKT/eNOS Signaling Pathway Due to Maternal GDM

**Figure 5:**
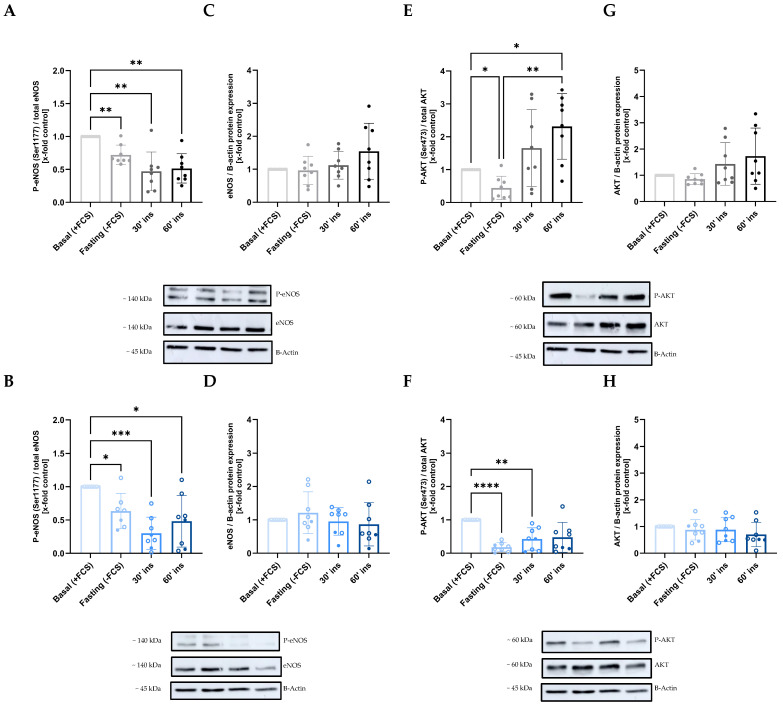
**Effect of maternal GDM on AKT and eNOS activation in HUVECs stimulated with insulin.** (**A**,**B**) eNOS activation, assessed as the p-eNOS/eNOS ratio, was not significantly increased in either NG (**A**) or GDM (**B**) HUVECs following insulin stimulation. (**C**,**D**) Total eNOS expression remained unchanged after insulin stimulation in both NG (**C**) and GDM (**D**) HUVECs. (**E**,**F**) AKT activation, measured as the p-AKT/AKT ratio, was significantly increased in NG (**E**) HUVECs after 60 min of insulin stimulation, but not in GDM (**F**) HUVECs. (**G**,**H**) Total AKT expression showed a non-significant increase in NG (**G**) HUVECs after 60 min of insulin stimulation, while no changes were observed in GDM (**H**) HUVECs. Data are presented as mean ± standard deviation. Statistical significance was determined using repeated measures one-way ANOVA, with *p* < 0.05 considered statistically significant. Asterisks indicate significance levels: * *p* < 0.05, ** *p* < 0.01, *** *p* < 0.001, **** *p* < 0.0001. Samples sizes were n_NG_ = 8 (gray scale) and n_GDM_ = 8 (blue scale) (with n_dGDM_ = 6 shown as empty dots and n_iGDM_ = 2 as filled dots). eNOS, endothelial nitric oxide synthase; GDM, gestational diabetes mellitus; NG, normoglycemic group; p-eNOS, phosphorylated eNOS.

Given the results indicating impaired NO-dependent vasodilation in fetoplacental vessels from GDM patients, we aimed to further investigate the underlying mechanisms at the endothelial level. We isolated human umbilical vein endothelial cells (HUVECs) from normoglycemic (NG) and GDM pregnancies and stimulated them with insulin to activate the AKT/eNOS pathway. As expected, serum starvation (absence of fetal calf serum, FCS) reduced eNOS phosphorylation at Ser1177 in both NG (Figure 5A) and GDM (Figure 5B) HUVECs. However, insulin stimulation for 30 min or 1 h did not result in increased eNOS phosphorylation in either group. Similarly, insulin had no significant effect on total eNOS expression in NG (Figure 5C) or GDM (Figure 5D) cells. Regarding AKT activation, we again observed a reduction in phosphorylation following serum starvation in both NG (Figure 5E) and GDM (Figure 5F) HUVECs. Notably, insulin induced a significant increase in AKT phosphorylation only in NG HUVECs after 60 min of stimulation. However, total AKT expression remained unchanged by insulin treatment in both NG (Figure 5G) and GDM (Figure 5H) cells.

Based on the observed impairment of insulin-stimulated AKT phosphorylation in GDM HUVECs despite unaltered eNOS expression and activation, we hypothesized that the endothelium in GDM may activate alternative vasodilatory pathways as compensatory mechanism. To investigate this, we analyzed the expression of key genes involved in different vasodilatory pathways in HUVECs treated with or without insulin for 24 h.

**Figure 6:**
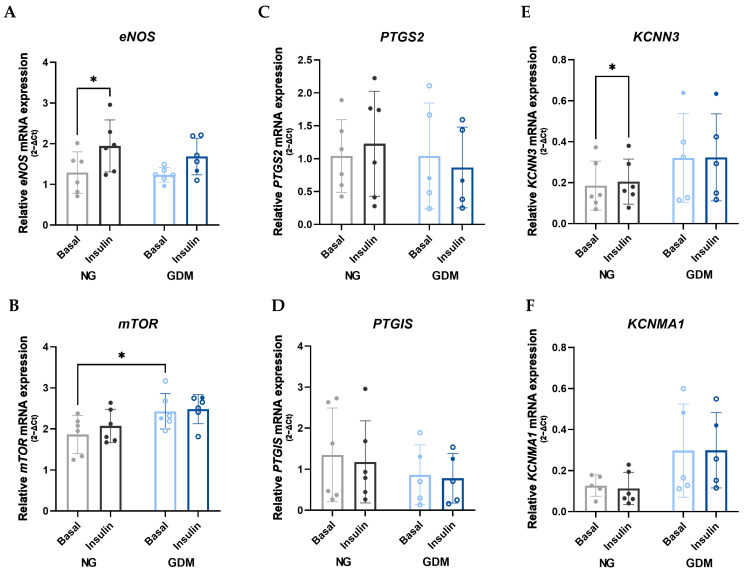
**Expression differences in vasodilation-related genes between NG and GDM HUVECs, with or without insulin stimulation.** (**A**) Insulin stimulation induces an upregulation of *eNOS* mRNA expression in NG HUVECs, but not in GDM HUVECs. However, no significant differences in *eNOS* expression are observed between NG and GDM groups under either condition. (**B**) *mTOR* expression is upregulated in GDM HUVECs under basal conditions. However, insulin stimulation does not affect either NG or GDM cells. (**C**,**D**) *PTGS2* and *PTGIS* mRNA expression, genes involved in the prostacyclin pathway, are not significantly affected by insulin treatment or by GDM status. (**E**,**F**) *KCNN3* and *KCNMA1*, genes involved in the endothelium-derived hyperpolarizing factor pathway, show a trend toward increased expression in GDM HUVECs compared to NG, although the differences are not statistically significant. *KCNN3* expression is significantly upregulated by insulin in NG HUVECs only. Data are presented as mean ± standard deviation. Statistical significance was determined using two-way ANOVA, with *p* < 0.05 considered statistically significant, indicated by an asterisk (*). Samples sizes were n_NG_ = 6 (gray scale) and n_GDM_ = 6 (blue scale) (with n_dGDM_ = 5 shown as empty dots and n_iGDM_ = 1 as filled dots). KCNMA1, potassium calcium-activated channel subfamily M alpha 1; KCNN3, potassium calcium-activated channel subfamily N member 3; mTOR, mechanistic target of rapamycin kinase; PTGIS, prostaglandin I2 synthase; PTGS2, prostaglandin-endoperoxide synthase 2.

Long-term insulin stimulation (24 h) induced an increase in eNOS mRNA expression in NG HUVECs, whereas this response was absent in GDM cells (Figure 6A), further supporting an impaired insulin-mediated AKT/eNOS pathway in GDM. In addition, we observed a significant increase in mechanistic target of rapamycin kinase (mTOR) mRNA expression in GDM HUVECs under basal conditions, compared to control, but no differences after insulin stimulation (Figure 6B). Analysis of prostacyclin pathway genes prostaglandin-endoperoxide synthase 2 (*PTGS2*) (Figure 6C) and prostaglandin I2 synthase (*PTGIS*) (Figure 6D), revealed no significant differences between NG and GDM HUVECs, nor any effect of insulin stimulation, suggesting that this pathway may not be differentially regulated in GDM.

Interestingly, expression of potassium calcium-activated channel subfamily N member 3 (*KCNN3*), a key component of the endothelium-derived hyperpolarizing factor (EDHF) pathway, showed no significant differences between NG and GDM groups. However, a modest but significant increase was observed in NG cells following insulin stimulation (Figure 6E). In contrast, potassium calcium-activated channel subfamily M alpha 1 (*KCNMA1*), another gene associated with EDHF-mediated responses, showed a trend toward increased expression in GDM HUVECs compared to NG, both under basal conditions (*p* = 0.1195) and after insulin stimulation (*p* = 0.0548) (Figure 6F), indicating a possible compensatory upregulation of the EDHF pathway in GDM.

## 3. Discussion

Maternal GDM is known to mediate both short- and long-term clinical complications in the mother and the offspring. The exact pathophysiology underlying the development of GDM remains incompletely understood. Established maternal risk factors such as advanced age, higher pre-pregnancy BMI, and excessive gestational weight gain contribute to increased insulin resistance and limited β-cell reserve [21,22]. Increasing evidence also suggests that fetal sex may influence maternal glucose metabolism, with studies reporting higher postprandial glucose levels and reduced β-cell function in pregnancies with male fetuses, resulting in a modest increase in GDM risk [23,24]. Accordingly, in our cohort, women with GDM had significantly higher BMI than controls. The exploratory analyses of the sex-stratified data (Supplementary Table S1) revealed that this increase was particularly pronounced in mothers carrying female fetuses. This suggests that additional maternal metabolic risk factors, such as elevated BMI, may be necessary to trigger GDM in these pregnancies. In contrast, male fetuses may impose an inherent metabolic challenge. These findings support the concept that maternal glucose regulation can be influenced by fetal sex [25]. However, the observed sex-specific effects might be affected by the small subgroup sizes in our cohort, potentially causing statistical fluctuations. Considering these maternal and fetal risk patterns, GDM not only affects pregnancy outcomes but also has a negative impact on the health of the offspring.

Infants born to mothers with GDM are at increased risk of delivery complications, neonatal hypoglycemia, reduced oxygenation, and respiratory difficulties [26]. Consistently, we observed a significant reduction in the 5 min Apgar score in the GDM group, a finding previously linked to increased placental weight [27]. This supports adverse GDM-related short-term clinical outcomes. As long-term consequences, children born to mothers with GDM are at an elevated risk of developing cardiovascular diseases (CVD) and type 2 diabetes later in life [26]. In line with the Developmental Origins of Health and Disease (DOHaD) hypothesis, intrauterine exposure to adverse environmental factors, such as maternal hyperglycemia, may disrupt the fetus’s normal development and increase the risk of different diseases later in life [28]. Within this context, the placenta plays a key role in allowing proper fetal growth and development. Thus, an inadequate placental function may predispose the offspring to future health conditions [29].

We observed that babies born to mothers with GDM are delivered earlier, and although their birth weights did not differ significantly from those of healthy pregnancies, their placentas were notably larger. Consequently, the placental coefficient, defined as the body weight to placental weight ratio (BW/PW), is reduced in the GDM group. These findings indicate a compensatory enlargement of placentas from GDM patients due to decreased placental efficiency. Moreover, they align with a large population-based study analyzing over 500,000 births from non-diabetic mothers and more than 8000 births from diabetic mothers (including type 1 and 2 diabetes (T1DM, T2DM) and GDM). That study reported a significant increase in placental weight, a reduced BW/PW ratio, and a lower mean gestational age at delivery in the diabetic group [30]. In our study, all GDM patients underwent cesarean delivery, which may partially explain the lower gestational age in the GDM group, as cesarean delivery is often indicated due to increased risks of adverse pregnancy outcomes and the presence of underlying comorbidities. Similarly, another study has found that placentas from obese and GDM pregnancies are larger and thicker [31], suggesting diminished placental efficiency in maternal-fetal exchanges [32].

Placental insufficiency during late gestation has been linked to the paracrine action of human placental lactogen (hPL or CSH) [33]. Both *CSH-1* (*hPL-A*) and *CSH-2 (hPL-B*) genes are expressed in the placenta, with *CSH-1* being primarily responsible for the majority of hPL protein expression [34]. We demonstrated in late pregnancy that placentas from mothers with GDM exhibit a significant upregulation of the *CSH-1* gene in the fetal-side vasculature. CSH-1 has insulin-antagonistic effects and contributes to maternal insulin resistance, ensuring increased glucose availability for the fetus, thereby regulating fetal growth [35,36]. This elevated expression may influence fetal metabolism by modulating insulin signaling pathways, potentially contributing to fetal hyperglycemia and thereby facilitating the development of fetal insulin resistance in GDM. In contrast, *CSH-2* does not show comparable upregulation, suggesting that CSH-1 specifically adapts to maternal metabolic alterations, while CSH-2 may play a role in maintaining placental metabolism. In the context of maternal hyperglycemia, the increased expression of hPL in fetal vessels may further exacerbate fetal hyperglycemia, which in turn could trigger fetal hyperinsulinemia as a compensatory response [37]. Prolonged hyperinsulinemia may eventually lead to insulin resistance in the offspring, a condition known to be associated with endothelial dysfunction and an increased risk of CVD [38].

When analyzing the effect of insulin on fetoplacental vessels, we observed a diminished vasodilation response in GDM. The interplay between insulin and nitric oxide (NO) in the regulation of vascular resistance has been extensively studied in other forms of diabetes [39], with reports dating back to the 1990s documenting impaired endothelium-dependent vasodilation in patients with insulin-dependent diabetes mellitus [40]. More recently, impaired insulin-mediated vasodilation has also been described in the microvasculature of women with GDM [41]. Recently, we reported endothelial dysfunction in GDM fetoplacental vessels characterized by reduced vasorelaxation in response to substance P. In addition, our recent study revealed a decrease in the mRNA expression of tachykinin receptor 1 (*TACR1*), the receptor for substance P [42]. The present study demonstrates a reduction in total insulin receptor *(INSR*) protein expression in arterial fetoplacental vessels of GDM patients, while the pINSR remained unchanged. This suggests that chronic hyperglycemia during gestation leads to downregulation of INSR, thus diminishing its signaling capacity and contributing to impaired insulin-mediated vascular responses. In support of this concept, Konishi and Sakaguchi [43] reported that mice with an endothelium-specific INSR knockout develop systemic insulin resistance, likely due to delayed insulin delivery and action in insulin-sensitive tissues such as skeletal muscle, brown adipose tissue, and specific brain regions. Conversely, endothelial overexpression of insulin receptor substrate-1 (IRS1) in ApoE^−^/^−^ mice enhanced insulin signaling and improved vascular function in the aorta [44], further highlighting the importance of endothelial insulin signaling in systemic metabolic regulation. However, it is important to notice that the ratio of phosphorylated to total INSR did not change between normoglycemic and GDM patients. Even while there is a downregulation of total INSR, all available receptors can still be equally activated.

Insulin induces vasodilation primarily through the activation of endothelial nitric oxide synthase (eNOS). However, in a context of endothelial insulin resistance, there is a selective decrease in insulin-mediated effects on the PI3K-AKT-eNOS pathway [45], causing an imbalance between NO and endothelin-1 production [46]. In our study, we observed that NG vessels treated with the nitric oxide synthase inhibitor L-NAME exhibited significantly reduced vasorelaxation in response to insulin compared to untreated vessels. This confirms that the insulin-mediated vasodilation is NO-dependent. However, this inhibitory effect of L-NAME was not observed in vessels from GDM patients, suggesting an impairment of the eNOS signaling pathway. This indicates that diminished insulin receptor availability in GDM compromises the NO-dependent component of insulin-mediated vasodilation, thereby linking altered receptor expression to impaired vascular function. Because most fetoplacental vessels were used for functional experiments, the remaining tissue was insufficient for further molecular analyses. Therefore, human umbilical vein endothelial cells (HUVECs) were used as a proof-of-concept model to study insulin signaling. Although the endothelium from placental arteries and the umbilical vein may differ in phenotype and function [47,48], both derive from the same fetoplacental circulation and thus provide complementary insights into endothelial function at the vascular and cellular levels. Accordingly, we examined insulin-induced activation and expression of two key downstream components of this pathway in HUVECs: AKT and eNOS. In NG HUVECs, insulin stimulation for 60 min resulted in a significant increase in AKT phosphorylation at Ser473, indicating AKT activation, along with a trend toward increased total AKT expression. In contrast, neither phosphorylated nor total AKT levels increased in GDM HUVECs, suggesting impaired activation of the PI3K pathway. Similarly, Wang et al. reported a reduced ratio of phosphorylated AKT to total AKT in HUVECs derived from insulin-treated GDM pregnancies [49]. The inactivation of the PI3K/AKT pathway has also been described in endothelial cells exposed to hyperglycemia, leading to endothelial dysfunction [50]. Consequently, hyperglycemia inhibits eNOS activity by decreasing eNOS phosphorylation at Ser1177, the AKT phosphorylation site [51]. Reduced eNOS phosphorylation and NO production have been reported in the offspring of diabetic rats [52] and reduced NO synthase activity in stem villous vessels of GDM placentas [53]. In our study, however, neither NG nor GDM HUVECs showed a significant increase in phosphorylated or total eNOS protein expression after insulin stimulation. Nevertheless, at the mRNA level, *eNOS* expression was increased in NG HUVECs after 24 h of insulin stimulation, an effect that was not observed in GDM cells. This indicates an impaired transcriptional activation of the NO pathway at the cellular level. These findings are in agreement with the functional measurements in placental arterial vessels, suggesting an early transcriptional impairment underlying the diminished NO-dependent vasodilatory response in GDM. We also detected increased *mTOR* expression in GDM HUVECs compared to NG under basal conditions, an adaptation known to negatively regulate the PI3K/AKT pathway and potentially contribute to further insulin resistance [54].

In this study, we demonstrate that the eNOS inhibition in GDM placental arterial vessels with L-NAME had no significant effect on insulin-induced relaxation. This indicates that the observed vasodilation is mediated by NO-independent mechanisms. An impaired PI3K/AKT pathway could lead to a shift activating the AMPK pathway [55] and increasing vasoconstriction [46]. Both prostacyclin (PGI_2_) and endothelium-derived hyperpolarizing factor (EDHF) represent important vasodilatory mechanisms in endothelial cells that function independently of the PI3K/AKT/eNOS pathway. Both pathways have been shown to be impaired under diabetic conditions. Studies using rats exposed to maternal diabetes during gestation have demonstrated decreased expression of the prostacyclin receptor and reduced vasodilation in the aorta [56]. In our study, however, we did not observe any significant differences in the mRNA expression of the genes *PTGS2* and *PTGIS*, which are involved in prostacyclin synthesis. As for EDHF, its function is usually compromised in GDM, primarily due to reduced endothelial calcium signaling and impaired potassium channel activity, which leads to diminished vasodilation, particularly in small resistance arteries [57]. In our study, we observed a non-significant trend toward increased expression of *KCNN3* and *KCNMA1* in GDM HUVECs compared to controls. This may reflect a compensatory response by the endothelium to counteract impaired PI3K/AKT signaling and partially preserve vasodilatory capacity through the recruitment of NO-independent pathways when insulin signaling is impaired.

## 4. Limitations

The cross-sectional nature of our study limits the ability to draw causal inferences and only provides a snapshot of placental and metabolic parameters at the time of delivery. While we standardized procedures as much as possible, some degree of variability is inherent to studies using human placental samples. Part of this variability could be attributed to certain confounding factors such as maternal blood pressure or lipid profile. However, due to the lack of access to these parameters, we did not adjust our analyses for any confounding factors. Even though we performed an exploratory analysis based on fetal sex differences, these analyses are likely underpowered given the rather small sample size. Some of the apparent sex-specific differences might not represent robust biological effects (see Supplementary Material). Similarly, due to the rather small sample size, we were unable to stratify the GDM group based on diet-treated or insulin-controlled GDM. This could have provided a greater insight into the impact of GDM treatment on fetoplacental vascular function and insulin signaling [42]. Nevertheless, the use of human tissue represents a major strength of our study, as it provides direct insight into human physiology that cannot be fully captured by animal or in vitro models.

## 5. Materials and Methods

### 5.1. Clinical Data and Tissue Collection

This study included placental and umbilical cord samples from 52 parturient women who delivered at the University Hospital Carl Gustav Carus in Dresden (Germany). Participants were classified into two groups based on the results of an oral glucose tolerance test (OGTT): 33 women with normoglycemia (NG) and 19 women diagnosed with gestational diabetes mellitus (GDM). The GDM group was further subdivided into patients treated with insulin (iGDM, n = 9) and those managed through dietary intervention alone (dGDM, n = 10). Gestational diabetes was defined according to standard diagnostic criteria: fasting plasma glucose > 5.1 mM, 1 h post-load glucose > 10.0 mM, or 2 h post-load glucose > 8.5 mM. Women with OGTT values within the normal reference range were classified as normoglycemic (NG).

Clinical data were collected for all participants, allowing the analysis of maternal risk factors and neonatal short-term outcomes. In addition, several placental parameters were recorded, including placental weight, length, and width, as well as the weight and length of the umbilical cord.

This study was approved by the Ethics Committee of the Technical University of Dresden (reference number EK 277–07-2018 (BO), renewed in 2022) and conducted in accordance with the ethical principles outlined in the Declaration of Helsinki.

### 5.2. Fetoplacental Vessels Isolation

The fetal side of the placenta, specifically the chorionic plate, was examined to identify and isolate fetal arterial vessels. The overlying amniotic membrane was carefully removed using anatomical forceps to expose the vessels. A 2 cm segment of each artery was then dissected from the underlying placental tissue, excised with surgical scissors, and placed in phosphate-buffered saline (PBS). Under a light microscope, residual connective tissue was carefully removed. To avoid mechanical compression, two to four vessel rings were precisely prepared using surgical scissors for subsequent vascular reactivity analyses in a Mulvany myograph system.

Additionally, fetal vessels approximately 1 cm in length were dissected from the basal maternal decidua, located deep within the chorionic villi. These vessels were also put in PBS and cleaned under the microscope, snap-frozen in liquid nitrogen, and stored at −80 °C for subsequent gene and protein expression analyses.

### 5.3. Mulvany Myograph

The Mulvany myograph (Multi Myograph System 620M, Danish Myo Technology, Aarhus, Denmark) was used to measure the contractile and relaxant responses of fetal placental arteries under controlled experimental conditions, allowing detailed assessment of vascular function.

Arterial rings were carefully mounted onto the pins of the myograph and placed into chambers filled with Krebs–physiological saline solution (Krebs–Henseleit) gassed with Carbogen and maintained at 37 °C. Passive stretch was applied to the vessels to establish their physiological resting length and baseline tension, allowing reproducible and comparable measurements of vascular responses. The maximal contractile capacity of each vessel segment was then determined by administering 80 mM potassium chloride (KCl). After thorough washing, vessels were pre-contracted using 10^−5^ M serotonin until a contraction plateau was reached. Insulin was then added cumulatively, starting at a concentration of 10^−9^ M and increasing stepwise to a final concentration of 3 × 10^−6^ M, and relaxation responses were recorded. Following another washout, the nitric oxide synthase (NOS) inhibitor L-NAME (300 mM) was added to the chamber and incubated for 30 min. The experimental protocol was then repeated.

### 5.4. Endothelial Cells Isolation and Culture

The umbilical cords obtained from NG and GDM pregnancies were used to isolate primary Human Umbilical Vein Endothelial Cells (HUVECs). Each umbilical cord was processed individually and incubated in a 0.5% collagenase II (Worthington Biochemical Corp., Lakewood, NJ, USA) solution at 37 °C to detach the endothelial cells from the vessel. Next, the single-donor cells were seeded in 2% gelatin-coated flasks and cultured in M199 medium supplemented with 10% fetal bovine serum (Capricorn Scientific, Ebsdorfergrund, Hessen, Germany), 100 mg/L streptomycin (Thermo Fisher Scientific, Waltham, MA, USA), 100,000 U/L penicillin (Thermo Fisher Scientific, Waltham, MA, USA), 500 µg/L amphotericin B (Thermo Fisher Scientific, Waltham, MA, USA), and 0.5% self-isolated retina calf eye growth factor. The cells were kept in culture at 37 °C in a humidified environment with 5% CO_2_ and used for experiments at passage 2.

### 5.5. Insulin Stimulation of Endothelial Cells

Confluent cells isolated from NG and GDM umbilical cords were stimulated with insulin to activate the AKT/eNOS signaling pathway. In one set of experiments, cells were treated with insulin (0.5 mg/mL; Sigma-Aldrich, Merck, Darmstadt, Germany) for 24 h. Subsequently, cells were lysed and RNA isolated. In a separate set of experiments, cells were serum-starved for one hour to reach a resting phosphorylation level. These cells were then stimulated with insulin (0.5 m mg/mL) for 30 min or 1 h to activate the pathway. Following stimulation, cells were washed with cold PBS containing phosphatase (PhosSTOP™ Roche, Basel, Switzerland) and protease inhibitors (Sigma-Aldrich, Merck, Darmstadt, Germany), and lysed for protein extraction.

### 5.6. RNA Isolation and RT-qPCR

To perform comparative gene expression analysis, RNA was isolated from both HUVECs and fetoplacental vessels. For HUVECs, we used the High Pure RNA isolation kit (Roche, Basel, Switzerland), following the manufacturer’s instructions. For the RNA isolation of the fetal vessels, the samples were mechanically homogenized using a Precellys homogenizer (VWR International GmbH, Erlangen, Germany) and RNA was then extracted using the peqGOLD TriFast RNA-Isolation Kit (VWR International GmbH, Erlangen, Germany), following the manufacturer’s protocol.

In both cases, the RNA concentration was determined using a NanoDrop™ One Microvolume UV-Vis Spectrophotometer (Thermo Fisher Scientific, Waltham, MA, USA). A total of 200 ng of RNA was reverse-transcribed into cDNA using SuperScript II Reverse Transcriptase (Invitrogen, Thermo Fisher Scientific, Waltham, MA, USA). The resulting cDNA was then used for quantitative PCR to evaluate differences in gene expression. The qPCR was performed using the Life Technologies 7500 Fast Real-Time PCR System (Thermo Fisher Scientific, Waltham, MA, USA) with Go Taq qPCR Master Mix (Promega, Madison, WI, USA) and the primers of interest (Table 2). The amplification program consisted of an initial denaturation step, followed by 40 cycles of further denaturation and annealing at 60 °C, and a melt curve stage. The specificity of the amplification was confirmed by a melting curve analysis. Relative gene expression was calculated using the ΔΔCt method, with TATA-Binding Protein (TBP) serving as the reference gene.

### 5.7. Protein Isolation and Western Blotting

HUVECs stimulated with insulin were lysed using ice-cold RIPA buffer supplemented with protease and phosphatase inhibitors. The lysates were centrifuged at 14,000× *g* for 10 min at 4 °C, and the supernatants containing soluble proteins were collected and stored at −80 °C until further analysis.

For fetal vessels, snap-frozen tissue samples were first manually grounded using a chilled mortar. Subsequently, 100 mg portions were homogenized using a Precellys homogenizer. Nuclear and cytosolic protein fractions were then isolated using the Active Motif Nuclear Extract Kit (Active Motif Europe, Waterloo, Belgium) according to the manufacturer’s instructions. Extracted fractions were stored at −80 °C for subsequent use. The resulting cytosolic fraction was employed for downstream protein analyses.

Protein concentrations of both cell and tissue lysates were quantified using the Micro BCA™ Protein Assay Kit (Thermo Fisher Scientific, Waltham, MA, USA) on a Fluostar Optima microplate reader (BMG Labtech GmbH, Ortenberg, Germany). Equal amounts of protein were loaded for Western blot analysis.

Primary antibodies used for Western blot analysis included phospho-insulin receptor (Tyr1150/1151) (1:1000 in 5% BSA in TBS-T; #04-299, Sigma-Aldrich, Merck, Darmstadt, Germany), insulin receptor (1:1000 in 5% non-fat dry milk in TBS-T; ab137747, Abcam, Cambridge, UK), β-Actin (1:10,000 in 5% non-fat dry milk in TBS-T; #4967S, Cell Signaling Technology, Danvers, MA, USA), and GAPDH (1:5000 in 5% non-fat dry milk in TBS-T; ab8245, Abcam, Cambridge, UK). Corresponding HRP-conjugated secondary antibodies were applied. Signal detection was performed using Western Lightning™ Enhanced Luminol Reagent Plus (PerkinElmer, Waltham, MA, USA) and visualized with the Amersham ImageQuant 800 imager (Cytiva, Marlborough, MA, USA). Protein band densities were quantified using ImageJ v1.54g (Fiji) software, with all values normalized to housekeeping proteins (β-Actin or GAPDH) used as loading controls.

### 5.8. Statistical Analysis

Statistical analysis was performed using GraphPad Prism 10 (GraphPad Software Inc., La Jolla, CA, USA). Normality was assessed using the Shapiro–Wilk test. Data with a Gaussian distribution are presented as mean ± standard deviation (SD), while non-normally distributed data are presented as median and interquartile range (IQR). Normally distributed data were analyzed using two-tailed unpaired *t*-tests or Welch’s *t*-tests (when variances were unequal), one-way ANOVA followed by Tukey’s multiple comparisons test, or two-way ANOVA for experiments involving two independent variables. Non-normally distributed variables were analyzed using the Mann–Whitney U test or Kruskal–Wallis test. Categorical variables were analyzed using Fisher’s exact test. Statistical significance was set at *p* < 0.05 for all analyses.

## 6. Conclusions

In summary, our study shows that maternal GDM impairs the fetoplacental insulin-induced vasodilation via the PI3K/AKT/eNOS pathway. This may contribute to adverse neonatal outcomes observed in GDM pregnancies, such as earlier delivery, larger placentas, reduced placental efficiency, and the increased long-term risk of developing metabolic and cardiovascular diseases in the offspring.

## Figures and Tables

**Table 1:** Clinical data of study participants.

	NG (n = 33)	GDM (n = 19)	*p*
**Maternal data**			
Age (years) (mean ± SD) Body Mass Index (kg/m^2^) (mean ± SD) Weeks of pregnancy (mean ± SD) Fasting glycemia (mM) (mean ± SD) Glycemia 1 h post glucose ingestion (mM) (mean ± SD) Glycemia 2 h post glucose ingestion (mM) (mean ± SD) Delivery mode (vaginal/cesarean)	33.68 ± 4.66 23.88 ± 5.20 39.23 ± 1.10 4.81 ± 0.70 6.71 ± 0.73 6.06 ± 1.04 9:24	36.00 ± 6.16 30.72 ± 8.44 38.46 ± 0.63 5.25 ± 0.54 9.94 ± 1.31 8.09 ± 1.24 0:19	0.1474 (ns) 0.0012 (**) 0.0122 (*) 0.0788 (ns) <0.0001 (****) 0.0058 (**) 0.0184 (*)
**Neonatal data**			
Sex (male/female) Birth weight (g) (mean ± SD) Apgar score 1 min (median (IQR)) Apgar score 5 min (median (IQR)) Apgar score 10 min (median (IQR))	21:12 3348.18 ± 403.57 9 (0) 10 (1) 10 (0)	11:8 3360.56 ± 624.48 9 (1) 9 (1.75) 10 (0)	0.7708 (ns) 0.9334 (ns) 0.4119 (ns) 0.0057 (**) 0.9752 (ns)
**Placental data**			
Placental weight (g) (mean ± SD) Placental width (cm) (mean ± SD) Placental length (cm) (mean ± SD) Placental area (cm^2^) (mean ± SD) Umbilical cord weight (g) (mean ± SD) Umbilical cord length (cm) (mean ± SD)	526.71 ± 103.84 16.74 ± 1.93 19.26 ± 2.81 253.59 ± 50.72 30.88 ± 11.53 39.52 ± 12.15	707.31 ± 128.09 18.53 ± 2.22 21.94 ± 3.80 323.47 ± 87.57 35.75 ± 15.05 39.80 ± 11.54	0.0008 (***) 0.0120 (*) 0.0230 (*) 0.0094 (**) 0.3553 (ns) 0.9401 (ns)

Section headings in bold indicate grouping of related clinical variables. Normally distributed variables are presented as mean ± SD and compared using an unpaired two-tailed *t*-test or Welch’s *t*-test when variances were unequal. Non-normally distributed variables are shown as median (IQR) and analyzed using the Mann–Whitney U test. Categorical variables were analyzed using Fisher’s exact test. Statistical significance was set at *p* < 0.05. Asterisks indicate significance levels: * *p* < 0.05, ** *p* < 0.01, *** *p* < 0.001, **** *p* < 0.0001. Abbreviations: Apgar, activity, pulse, grimace, appearance, respiration; GDM, gestational diabetes mellitus; IQR, interquartile range; NG, normoglycemic group; ns, not significant.

**Table 2:** List of primers.

Target Gene	Primer Name	Amplicon Size	Sequence (5′ ⟶ 3′)
Chorionic somatomammotropin hormone 1 (*CSH-1* or *hPL-A*)	*CSH-1* forward	139	ACTGCTCAAGAACTACGGGC
	*CSH-1* reverse	139	AGGGGTCACAGGATGCTACT
Chorionic somatomammotropin hormone 2 (*CSH-2* or *hPL-B*)	*CSH-2* forward	81	CATCCTGTGACCGACCCCT
	*CSH-2* reverse	81	TATTAGGACAAGGCTGATGGGC
Endothelial Nitric Oxide Synthase	*eNOS* forward	146	GAACCTGTGTGACCCTCACCCC
	*eNOS* reverse	146	TGGCTAGCTGGTAACTGTGC
Mammalian target of rapamycin	*mTOR* forward	137	GGCCATCCGGGAATTTTTGT
	*mTOR* reverse	137	TCGTGCTCTGAATTGAGGTGT
Potassium Calcium-Activated Channel Subfamily M Alpha 1	*KCNMA1* forward	527	GGTGTTGGGTGAGTTCC
	*KCNMA1* reverse	527	TCTCCAGTGCCTTCGTG
Potassium Calcium-Activated Channel Subfamily N Member 3 (SKCa3)	*KCNN3* forward	174	GTTCCATCTTGACGCTCCTC
	*KCNN3* reverse	174	TGGACACTCAGCTCACCAAG
Prostaglandin I2 Synthase	*PTGIS* forward	103	GGGATCTCCACATCTGCGTT
	*PTGIS* reverse	103	ACTGCCTGGGGAGGAGTTAT
Prostaglandin-Endoperoxide Synthase 2	*PTGS2* forward	223	GTTGGTGGCGGTGACTTGTT
	*PTGS2* reverse	223	AGATCATAAGCGAGGGCCAG
TATA-Binding Protein	*TBP* forward	133	CGCCGGCTGTTTAACCTTCG
	*TBP* reverse	133	AGAGCATCTCCAGCACACTC

## Data Availability

The data supporting the conclusions of this article will be made available by the authors on request.

## Supplementary Materials

The following supporting information can be downloaded at: https://www.mdpi.com/article/10.3390/ijms262311507/s1.

## Abbreviations

The following abbreviations are used in this manuscript:

Apgar	Activity, pulse, grimace, appearance, respiration
ARDS	Acute respiratory distress syndrome
CSH-1, 2	Chorionic Somatomammotropin Hormone 1, 2
CVD	Cardiovascular diseases
GAPDH	Glyceraldehyde-3-phosphate dehydrogenase
EDHF	Endothelium-derived hyperpolarizing factor
eNOS	Endothelial Nitric Oxide Synthase
GDM	Gestational Diabetes Mellitus
INSR	Insulin Receptor
IQR	Interquartile range
KCNMA1	Potassium calcium-activated channel subfamily M alpha 1
KCNN3	Potassium calcium-activated channel subfamily N member 3
mTOR	Mechanistic Target Of Rapamycin kinase
NG	Normoglycemic
NO	Nitric Oxide
OGTT	Oral Glucose Tolerance Test
p-eNOS	Phosphorylated endothelial Nitric Oxide Synthase
p-INSR	Phosphorylated Insulin Receptor
PTGIS	Prostaglandin I2 synthase
PTGS2	Prostaglandin-endoperoxide synthase 2
SD	Standard Deviation
