# Supplementary material for: Maternal Gestational Diabetes Impairs Fetoplacental Insulin-Induced Vasodilation via AKT/eNOS Pathway and Reduces Placental Efficiency
Source: Int J Mol Sci. 2025 Nov 27;26(23):11507. doi: 10.3390/ijms262311507 (PMC12692426; doi:10.3390/ijms262311507)
Supplement: Supplementary file 1 [file ijms-26-11507-s001.zip › ijms-3947902-supplementary.pdf]

Table S1. Clinical data of study participants, stratified by fetal sex.

	Male NG	Male GDM	Female NG	Female GDM
**Maternal data**				
Age (years) (mean ± SD)	32.95 ± 4.05	34.13 ± 5.64	34.85 ± 5.29	38.25 ± 6.26
Body Mass Index (kg/m^2) (mean ± SD)	23.27 ± 4.58	27.32 ± 5.93	24.21 ± 5.70	34.49 ± 9.41 (**)
Weeks of pregnancy (mean ± SD)	39.27 ± 0.73	38.34 ± 0.83 (*)	39.23 ± 1.49	38.61 ± 0.27
Fasting glycemia (mM) (mean ± SD)	4.70 ± 0.41	5.31 ± 0.41	4.95 ± 0.90	5.17 ± 0.21
Glycemia 1 h post glucose ingestion (mM) (mean ± SD)	6.46 ± 0.52	9.99 ± 1.14 (****)	7.40 ± 0.79	9.89 ± 1.49 (**)
**Neonatal data**				
Birth weight (g) (mean ± SD)	3394.76 ± 397.36	3751.88 ± 517.22 (*, #)	3259.23 ± 386.53	3284.38 ± 299.57 (#)
Apgar score 1 minute (median (IQR))	9(0)	9(0) (#)	9(0)	8(0.5) (**, #)
Apgar score 5 minutes (median (IQR))	10(1)	9(0.25)	9(1)	9(0.75) (*)
Apgar score 10 minutes (median (IQR))	10(0)	10(0) (#)	10(1)	10(1) (#)
**Placental data**				
Placental weight (g) (mean ± SD)	587.86 ± 107.99	749.50 ± 146.66 (**)	522.08 ± 81.79	665.13 ± 88.01 (**)
Placental coefficient (g) (mean ± SD)	5.87 ± 0.80	5.07 ± 0.50 (*)	6.32 ± 0.75	4.99 ± 0.46 (***)
Umbilical cord weight (g) (mean ± SD)	40.25 ± 12.57	40.38 ± 9.82	38.38 ± 11.40	39.14 ± 13.21
Umbilical cord length (cm) (mean ± SD)	30.82 ± 11.23	37.11 ± 17.61	27.83 ± 10.19	28.33 ± 2.49

Normally distributed variables are reported as mean ± standard deviation (SD), whereas non-normally distributed variables are presented as median (interquartile range, IQR). Statistical significance was assessed using a two-way ANOVA, with factors sex and metabolic condition. A significance threshold of $p < 0.05$ was applied. Significant differences between NG vs. GDM within the same sex (NG male vs. GDM male; NG female vs. GDM female) are indicated by (*), while significant differences between males and females within the same metabolic condition (NG male vs. NG female; GDM male vs. GDM female) are indicated by (#). Abbreviations: Apgar, activity, pulse, grimace, appearance, respiration; GDM, gestational diabetes mellitus; IQR, interquartile range; NG, normoglycemic group; ns, not significant. * $p < 0.05$; ** $p < 0.01$; *** $p < 0.001$; **** $p < 0.0001$.
